# Oral Sampling of Little Brown Bat *(Myotis lucifugus*) Maternity Colonies for SARS-CoV-2 in the Northeast and Mid-Atlantic, USA

**DOI:** 10.3390/ani13040550

**Published:** 2023-02-04

**Authors:** Megan L. Moran, William Boyd, Jesse L. De La Cruz, Andrea S. Bertke, W. Mark Ford

**Affiliations:** 1 Department of Fish and Wildlife Conservation, Virginia Polytechnic Institute and State University, Blacksburg, VA 24061, USA; 2 Virginia-Maryland College of Veterinary Medicine, Virginia Polytechnic Institute and State University, Blacksburg, VA 24061, USA; 3 Department of Population Health Sciences, Virginia-Maryland College of Veterinary Medicine, Virginia Polytechnic Institute and State University, Blacksburg, VA 24061, USA; 4 Center for Emerging Zoonotic and Arthropod-Borne Pathogens, Blacksburg, VA 24061, USA; 5 U.S. Geological Survey, Virginia Cooperative Fish and Wildlife Research Unit, Blacksburg, VA 24061, USA

**Keywords:** SARS-CoV-2, coronavirus, little brown bats, *Myotis lucifugus*

## Abstract

**Simple Summary:**

A wide variety of coronaviruses are enzootic in bats worldwide. SARS-CoV-2, the virus responsible for the COVID-19 pandemic, is suspected to have originated in horseshoe bats (*Rhinolophidae* spp.) in Asia, though the spillover event is unknown. SARS-CoV-2 has not been detected in wild North American bats at the time of this submission, although it has been detected in other native wildlife, such as white-tailed deer (*Odocoileus virginianus*). Further interspecies transmission may establish new viral reservoirs and mutations which may negatively impact wildlife, livestock, and human health. The potential introduction of SARS-CoV-2 into North American bat populations is of interest to wildlife managers due to recent declines of several species, including little brown bats (*Myotis lucifugus*), which have suffered large population collapse due to white-nose syndrome (WNS). We analyzed saliva samples from 235 individual little brown bats from a total of eight maternity colonies throughout the Northeastern and Mid-Atlantic United States. No bat tested positive for SARS-CoV-2 by RT-qPCR, indicating the virus is either not present or that it persists in undetectable levels in little brown bat populations in this region.

**Abstract:**

The potential introduction of SARS-CoV-2, the virus responsible for the COVID-19 pandemic, into North American bat populations is of interest to wildlife managers due to recent disease-mediated declines of several species. Populations of little brown bats (*Myotis lucifugus*) have collapsed due to white-nose syndrome (WNS), a disease caused by the introduction and spread of the fungal pathogen *Pseudogymnoascus destructans* (*Pd*). Throughout much of the United States and southern Canada, large colonies of the species routinely established diurnal roosts in anthropogenic structures, thereby creating the potential for direct human contact and cross-species disease transmission. Given recent declines and the potential for further disease impacts, we collected oral swabs from eight little brown bat maternity colonies to assess the presence and prevalence of SARS-CoV-2 by RT-qPCR analysis. Little brown bat colonies in Maryland (*n* = 1), New Hampshire (*n* = 1), New Jersey (*n* = 2), New York (*n* = 1), Rhode Island (*n* = 2), and Virginia (*n* = 1) were taken during May-August, 2022. From 235 assayed individuals, no bat tested positive for SARS-CoV-2. Our results indicate that little brown bats may not contract SARS-CoV-2 or that the virus persists at undetectable levels in populations of the Mid-Atlantic and Northeast during summer months. Nonetheless, continued monitoring and future work addressing other seasons may still be warranted to conclusively determine infection status.

## 1. Introduction

Chiroptera, the order including all bat species, contains > 1400 described species and represents approximately 20% of all mammalian diversity on the planet [1]. Bats are an important biodiversity component and provide numerous ecosystem services including insect control, pollination and seed dispersal [2,3]. Genetic analysis has dated the origin of bats to the Eocene, approximately 50–52 million years ago [4]. The high diversity and long evolutionary history of Chiroptera has allowed the taxon to serve as a reservoir for many viral pathogens, including coronaviruses, which have demonstrated spillover into humans and other vertebrates [5,6,7]. Flight allows high vagility, and many species migrate annually over long distances, contributing to spread intra- and inter-specifically between bat populations and other mammalian taxa. Similarly, social gregariousness of many bat species also can contribute to intra- and inter-species pathogen persistence and spread [8,9].

The study of coronaviruses and potential spillover from certain bat species has been a priority since the 2002 epidemic of SARS-CoV-1, which likely spilled over from palm civets (*Paradoxurus hermaphroditus*) or raccoons (*Procyon lotor*) into human populations, though may have had ancestral origins in the *Rhinolophus* genus [9,10,11]. The need for and intensity of coronavirus research has increased more since the COVID-19 pandemic caused by the virus SARS-CoV-2 [7,12,13]. A wide variety of coronaviruses are enzootic in bats worldwide [7]. There are two mammalian coronavirus types, alpha and beta, which have been detected in 14 of 21 bat families [7,14]. SARS-CoV-2, a beta coronavirus, is suspected to have originated in horseshoe bats (*Rhinolophidae* spp.) in Asia [9,10,11,12,15], although the spillover event is currently unresolved [16]. This spillover event could have occurred through transmission from bat to human or alternatively, from bat to another species, which then spilled over into humans. The possibility of human transmission to an animal species, establishing a new viral reservoir, has long been a public health concern [17]. 

At present, SARS-CoV-2 has not been detected in wild North American bats, suggesting that spillover from humans has not occurred [18], as it has with other domestic and wild animals in close proximity to humans [19,20,21,22,23,24,25]. For example, ferrets (*Mustela furo*) and cats (*Felis catus*) have been found to be permissive to SARS-CoV-2 infection with cats demonstrating susceptibility to airborne infection [21]. Farmed mink (*Neovison vison*) also are susceptible to the SARS-CoV-2, exhibit viral pathologies, and have been documented to transmit the virus back to human populations [23,24]. More notably, white-tailed deer (*Odocoileus virginianus*) across multiple Midwestern states in the United States have a 30–50% seropositivity rate and have been found to shed virus in oronasal secretions, demonstrating that the virus is capable of transmission to and spread within a wildlife species that dwells in close proximity to or routinely comes into contact with humans [20]. Observational surveillance of white-tailed deer in Canada identified a divergent lineage of SARS-CoV-2, which was linked to human infection after adaptation in the deer, providing evidence for evolution in wildlife and subsequent transmission back to human populations [22]. 

Throughout much of the United States and southern Canada, two species of bats routinely day-roost in the non-hibernating spring through early fall seasons in aggregations in anthropogenic structures and come into direct and indirect contact with humans: the big brown bat (*Eptesicus fuscus*) [26] and the little brown bat (*Myotis lucifugus*) [27]. Extant little brown bat maternity colonies, typically comprised of >20–500 members, often use houses, attics, barns, bridges, tree crevices, and artificial roosts as day-roosts in close proximity to human habitation or activity [28]. In the summer, males and non-reproductive females typically day roost in trees, buildings, rock crevices, or wood piles [27]. In the winter, females and males congregate in hibernation sites, such as caves or abandoned mines, which can range in size from 10s to 100,000s of individuals [27,29]. Mating typically occurs during the swarming period before hibernation, when individuals from various breeding groups join together [27,29,30,31]. Any of these seasons could represent opportunity for disease transmission. Although laboratory inoculation of SARS-CoV-2 to big brown bats failed to produce infection [32], the susceptibility of most North American bat species, including the little brown bat, is unknown. The angiotensin converting enzyme 2 (ACE2) expressed in little brown bats was reported to efficiently bind SARS-CoV-2 spike protein and is abundantly expressed in the bat trachea and intestines, supporting the potential for host susceptibility [33]. 

For wildlife managers, the introduction of a novel virus for bats with yet unknown pathologies is troubling due to the prevalence of other contemporary stressors on many bat species [34,35,36,37]. Notably in eastern North America, these include wind-energy development impacts on non-hibernating, migratory bat species, and white-nose syndrome (WNS) in hibernating species. WNS, first documented in a little brown bat hibernaculum in upstate New York in 2006, is caused by the fungus *Pseudogymnoascus destructans* (*Pd*), which creates lesions on the wing, face, and ear membranes of bats during hibernation [38,39]. Because the disease causes arousal from torpor, the energetic demand for many infected bats is too high to survive the winter, which has led to large population declines of cave-hibernating bats in North America [38,40]. Since its initial discovery, WNS has spread to 38 US states and seven Canadian provinces and *Pd* has been documented in 18 bat species, 12 of which were confirmed to have WNS [40,41]. 

Mortality of the estimated several million little brown bats in eastern North America has approached 90% since the advent of WNS [40]. This level of mortality has led to their classification as an imperiled species by the International Union for Conservation of Nature (IUCN) red list and is currently under review for U.S. Federal endangered species status by the U.S. Fish and Wildlife Service (USFWS) [42,43]. The occurrence and spread of WNS also has led to intriguing observations whereby WNS infected bats display significantly higher levels of a naturally occurring bat coronavirus, *M. lucifugus* coronovirus (Myl-CoV), with a 60-fold increase in viral RNA in the intestines as compared to non-WNS infected bats [44]. Furthermore, the quantity of Myl-CoV correlated with the severity of WNS pathology. The authors suggest that this increase in viral replication can lead to an increase in viral shedding and could therefore result in higher infection rates in the population or pose a greater risk of spillover to susceptible species [44]. Therefore, the dormant, hibernating season occurrence of WNS in bats and subsequent physiological demands from fungal clearing and physiological repair may represent an opportunity for infection with SARS-CoV-2 due to immunomodulation [44,45]. If infected with SARS-CoV-2, adaptation and evolution of the virus could also be facilitated by WNS due to alterations in replication kinetics. Combined with their propensity for roosting in structures in close proximity to humans, SARS-CoV-2 infection in little brown bats could potentially lead to new spillover events of divergent viral lineages to humans, sustaining the pandemic.

Prior to the advent of WNS, large colonies of little brown bats routinely established diurnal roosts in anthropogenic structures in the Northeast and Mid-Atlantic portion of the United States, creating the potential for direct human contact and cross-species disease transmission. Accordingly, this species might be a potential viral reservoir with considerable implications to human health and further bat population decline [5,46]. Due to the current vulnerability of little brown bat populations, monitoring this species for SARS-CoV-2 could be vital for their management and future conservation. Given recent declines of the little brown bat and possible implications for public health, our objective was to sample several of the remaining, large maternity colonies of little brown bats, associated with human structures or use areas, for the presence and prevalence of SARS-CoV-2 in the Northeast and Mid-Atlantic.

## 2. Materials and Methods

### 2.1. Study Area

**Figure 1:**
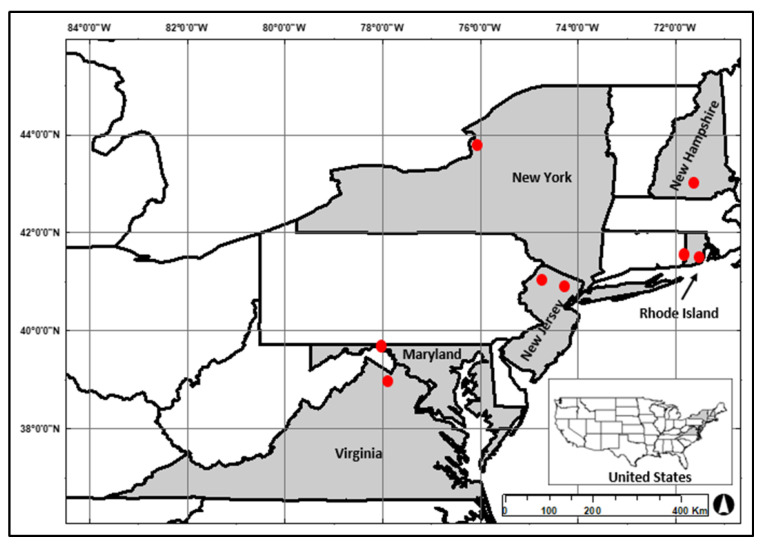
Little brown bat (*Myotis lucifugus*) capture locations, represented by red points, in the Northeast and Mid-Atlantic USA, 2022.

**Figure 2:**
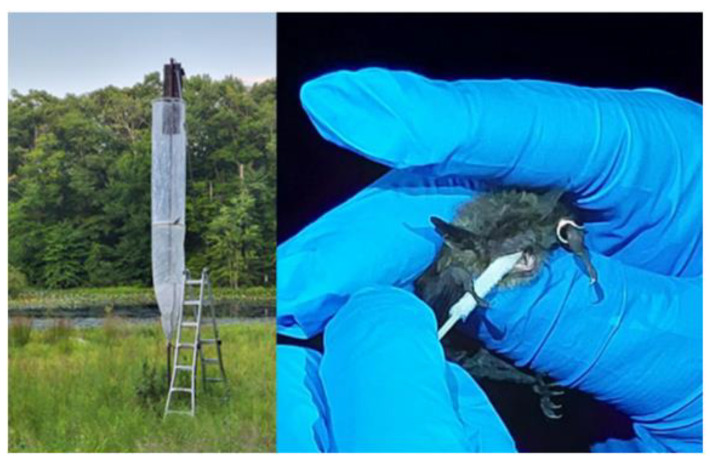
Left: Example of trap set up at a maternity colony in a bat box in New Jersey, 2022. Right: Oral swab collection from a little brown bat (*Myotis lucifugus*). Photo credit: J. De La Cruz.

Eight little brown bat maternity colonies were sampled in the Eastern Temperate Forest Biome [47] of the Northeast and Mid-Atlantic United States. Maternity colonies were captured on both public and private property: Maryland (*n* = 1), New Hampshire (*n* = 1), New Jersey (*n* = 2), New York (*n* = 1), Rhode Island (*n* = 2), and Virginia (*n* = 1) (Figure 1). All colonies were located in anthropogenic roosts, i.e., houses, barns, or artificial roosts (Figure 2). These colonies contained between ~ 20 to >300 individual little brown bats. 

### 2.2. Sample Collection

Little brown bat maternity colonies were identified with the assistance of cooperating state and Federal wildlife agency personnel. Each colony was sampled once, with the exception of the Maryland colony, which was sampled three separate times (once in May and twice in June 2022) due to a separate project that required multiple rounds of netting. New Hampshire, New York, Rhode Island, and Virginia colonies were sampled in June 2022. New Jersey colonies were sampled in August 2022. Bats were captured at evening colony emergence using a variety of mist nets and harp traps depending on the roosting structure (Figure 2). U.S. Fish and Wildlife Service-mandated personal protection equipment, i.e., N95 particulate respirators (3M Corporation, St. Paul, MN, USA (Any use of trade, firm, or product names is for descriptive purposes only and does not imply endorsement by the U.S. Government)) and disposable nitrile gloves (ThermoFisher Scientific, Waltham, MA, USA), were used to remove bats from traps or nets and to process each individual for body measurements and sample collection. Gloves were changed between each bat to avoid potential cross contamination. Species, body mass (g), sex, reproductive status (pregnant, lactating, post-lactating, testes descended and non-reproductive), wing score (0–3) from WNS damage [48], and age class (adult or juvenile) based on the degree of epiphyseal-diaphyseal fusion [49] were recorded for all captured bats (Table 1). For future identification and to benefit ongoing, long-term population monitoring, each bat was fitted with a uniquely numbered 2.9 mm aluminum alloy band (Porzana, East Sussex, UK), unless previously banded. Saliva samples were collected by swabbing the oral cavity with a pediatric nasopharyngeal swab (ThermoFisher Scientific, Waltham, MA, USA), allowing the bats to chew on the swab tip for at least 10 s (Figure 2). These swab tips were placed into storage vials containing 1 mL of DNA/RNA Shield (Zymo Research, Irvine, CA, USA) and stored on ice until transported to the laboratory, where they were stored at −80℃ until RNA extraction. 

### 2.3. RNA Extraction

Swab samples were thawed on ice and vigorously vortexed to distribute any material collected in the swab. 300 µL of sample were transferred to a new vial with 300 µL TRIzol LS (ThermoFisher Scientific, Waltham, MA, USA). Sample was vortexed and 200 µL of chloroform (Sigma-Aldrich, St. Louis, MO, USA) was added. Vials were inverted continuously for two minutes, incubated at room temperature for three minutes, and centrifuged for 15 min at 4 °C and 13,000 rpm. The aqueous layer was transferred to a new vial for RNA isolation. RNA was precipitated from the aqueous phase with 200 µL isopropanol and 0.5 µL glycogen, inverted several times to mix, and incubated at −80 °C overnight. Vials were centrifuged for 10 min at 4 °C and 13,000 rpm, supernatant was removed and discarded, the RNA was washed with 75% nuclease-free ethanol and then the RNA was resuspended in 20 µL of nuclease-free water. The RNA concentration of each sample was immediately determined by spectrophotometry using a NanoDrop™ 2000 spectrophotometer (ThermoFisher Scientific) and directly quantified by RT-qPCR assay. 

### 2.4. RT-qPCR for SARS-CoV-2

Reverse transcription quantitative PCR (RT-qPCR) was performed with iTaq Universal Probes One-Step Reaction Mix (Bio-Rad Laboratories Inc., Hercules, CA, USA), according to the manufacturer’s recommendations, on a Viia7 thermocycler (Applied Biosystems, Waltham, MA, USA). Primers and probe were specific for SARS-CoV-2 nucleoprotein RNA (2019-nCoV_N1 (IBFQ), Integrated DNA Technologies, Coralville, IA, USA), and each assay included eukaryotic 18S rRNA primer/probe mix (ThermoFisher Scientific) to quantify host RNA. Each assay included 5 µL 2X Reaction Mix, 0.25 µL RT, 0.75 µL primer/probe mix, 0.5 µL 18S rRNA primer/probe mix and 4 µL bat RNA sample. All plates included positive (2019 nCoV_N Positive Control) and negative (no RNA template) controls, and thresholds were adjusted to maintain consistency. The run method was 10 min 50 °C, 2 min 95 °C, followed by 40 cycles 3 s 95 °C and 30 s 55 °C.

## 3. Results and Discussion

A total of 235 little brown bats were captured from eight maternity colonies across six states in in the Northeast and Mid-Atlantic United States. Oral swabs were collected from each bat and screened for SARS-CoV-2 using an RT-qPCR assay specific for the viral nucleocapsid gene. All bats in our survey tested negative for the virus. Sensitivity of the assay and quantification of expression of the *18S rRNA* gene for each bat was sufficient to detect as few as eight copies of the viral genome. These results indicate that little brown bats have not yet contracted the virus, that it persists in undetectable levels, or has occurred outside our sampling time and infections have cleared in populations of the Northeast and Mid-Atlantic. 

Due to the current status of little brown bat populations, these findings are, thus far, encouraging from both the conservation and human health perspective. However, spillover is a function of exposure, and wildlife professionals that work with bats are recommended to follow protocols intended to decrease the likelihood of transmission [17,18,45]. Initial modeled estimates of susceptibility based on expert-opinion risk assessments predicted a range of likelihoods between 0.01–0.20 for little brown bats to potentially become infected by humans during summer fieldwork if no personal protective equipment precautions were taken [45]. Updated, median estimates of these initial models indicate that 0.83, 1.56, and 0.47 individuals per 1000 little brown bats could become infected with SARS-CoV-2 when exposed during research/monitoring, rehabilitation, and other encounters with infected humans, respectively [18]. Although estimates of human transmission to little brown bats are low, these results suggested a 33% probability of spread within bat populations given SARS-CoV-2 infection [17]. 

Susceptibility of little brown bats to SARS-CoV-2 infection is not fully known. Resistance seems to vary among bat species and families [32,50,51]. Seven of nine Egyptian fruit bats (*Rousettus aegyptiacus*) that were experimentally inoculated with the virus exhibited transient signs of infection, with virus detectable in the nasal cavity, trachea, lung, and lymphatic tissue at 4 days post-infection. Additionally, the virus was passed on to one of the three uninoculated contact bats in the study [51], indicating that transmission within the species was possible, although the R naught (R0) was low. Approximately half of experimentally inoculated Mexican free-tailed bats (*Tadarida brasiliensis*) showed evidence of viral shedding for up to 18 days [50]. However, these bats did not infect uninoculated contact animals, were able to clear the virus by the end of three weeks, and exhibited no clinical signs of the disease. More relevant to our study, big brown bats appear to be resistant to SARS-CoV-2, with experimentally-inoculated individuals exhibiting no signs of infection, viral excretion, transmission, or detectable virus in tissues [32].

Several Old World *Myotis* species harbor both the alpha and beta coronaviruses, although SARS-CoV-2, specifically, has not been detected in these species [52]. North American bat species have not been associated with other known naturally occurring beta coronaviruses [46]. Because little brown bats and big brown bats are both in the Family Vespertilionidae and are more closely related to each other than they are to free-tailed bats in the Family Molossidae [26,27,53], it is possible that little brown bats also will be resistant to the virus. Still, considering the small sample sizes in these laboratory viral-inoculation studies in bats, further research may clarify our understanding of the vulnerability of *M. lucifugus* to SARS-CoV-2. Continued surveillance is, therefore, advised, but with appropriate precautions. 

It is important to also note the potential effects of co-infection. An expert panel estimated that bats infected by *Pd* and exhibiting symptoms of WNS may be more susceptible to SARS-CoV-2 infection [45]. Additionally, because of this increased susceptibility and the elevated respiration rate caused by WNS, an infection risk model suggests that the risk of transmission to bats in hibernacula affected by WNS is approximately twice as high as for those without WNS [45]. Coinfection of WNS and SARS-CoV-2 could lead to increased viral replication, as exhibited in little brown bats when coinfected with WNS and their naturally occurring coronavirus, Myl-CoV [44]. That said, although the bats in our study showed low wing-scores forWNS-induced lesions and evidence of WNS infection in the preceding hibernation period, by the time of our summer surveys, infections were cleared and tissue repair had largely occurred. Still, because of this increased vulnerability during hibernation, reduction of other stressors during the winter hibernation period, i.e., due to human disturbance, become even more critical. 

Because little brown bats with WNS may be more susceptible to infection with SARS-CoV-2, the possibility exists that the virus could go undetected in maternity colonies, as infected individuals might either perish during hibernation or recover by summer [44,54]. A longitudinal study design testing maternity and bachelor colonies throughout the non-hibernation period (April to October), along with targeted sampling at hibernacula, could add clarity as to the presence of SARS-CoV-2 in this species regionally. If any bats were to be found infected with SARS-CoV-2, sequencing of the virus and correlation with presence and severity of WNS may also provide an indicator of the potential for adaptation and evolution of the virus in little brown bats. 

Protecting maternity colonies of little brown bats is of the utmost importance to long-term viability as the species continues to make, at best, only modest population increases (5–10%) following the long-running WNS-induced mortality event [28,54,55,56,57,58]. Results here, while regionally specific and limited in scope, indicate that little brown bat maternity colonies may not serve as reservoirs for SARS-CoV-2 and therefore likely do not represent a threat for human infection [18,59,60]. However, expanding both sample size and time period of surveillance would be prudent. In order to best protect bat and human health, however, following the guidelines set forth by Cook et al. (2022) [18] are essential; that is negative COVID-19 tests and vaccinations prior to bat interactions, as well as the proper use of N95 respirators when working directly with bats or in close proximity to bats are critical.

## 4. Conclusions

Our results indicate that SARS-CoV-2 is not currently present in the eight maternity colonies of little brown bats we sampled in the Northeast and Mid-Atlantic, United States. However, we cannot exclude the possibility that it persists at undetectable levels. Currently, little brown bats in this region likely do not represent a threat of viral transmission to humans. Nonetheless, because our work is preliminary and limited in temporal duration, transmission from humans to bat populations may still be possible if infected individuals are in close contact with bats, without taking recommended precautions to protect the bats. Accordingly, use of personal protective equipment, and COVID-19 vaccination and testing prior to interactions with bats would provide safety measures for individuals working in close contact with little brown bat colonies. Future work addressing other seasonal infectivity or using serologic approaches may still be warranted to conclusively determine disease status in this species and/or the relationship to stressors such as WNS.

## Figures and Tables

**Table 1:** *Myotis lucifugus* capture records from six states in the Northeast and Mid-Atlantic, USA, 2022.

Location	Female	Pregnant	Lactating	Male	Avg. Weight (g)	Avg. Forearm Length (mm)	Avg. Wing Score
Maryland	25	44%	0%	17	8.14 (1.70)	37.10 (1.25)	0.33 (0.57)
New Hampshire	31	10%	87%	0	8.36 (0.86)	38.54 (0.98)	0.03 (0.18)
New Jersey East	25	0%	0%	1	7.80 (0.76)	38.25 (0.93)	0.04 (0.20)
New Jersey West	12	0%	0%	0	8.01 (1.02)	37.77 (1.05)	0.08 (0.29)
New York	33	39%	56%	0	9.15 (1.61)	37.72 (1.13)	0.27 (0.45)
Rhode Island East	40	55%	42%	0	9.00 (1.35)	38.01 (1.10)	0.16 (0.37)
Rhode Island West	20	5%	71%	1	7.85 (0.92)	38.12 (1.04)	0.10 (0.30)
Virginia	27	0%	70%	3	7.49 (1.94)	36.41 (1.27)	0.06 (0.25)

Standard deviation is represented in parentheses.

## Data Availability

Data are available from the U.S. Geological Survey at https://www.sciencebase.gov/catalog/item/63a34fb9d34e176674f52154, accessed on 1 December 2022.
